# Part 1: A Systematic Review to Describe Existing Cultural Adaptations in Lifestyle, Nutrition, and Physical Activity Programs for Native Hawaiian, CHamoru, and Filipino Populations

**DOI:** 10.3390/ijerph22111673

**Published:** 2025-11-04

**Authors:** Monica K. Esquivel, Kristi Hammond, Bernice C. Delos Reyes, Dareon C. Rios, Niza Mian, Elaine C. de Leon, Samantha M. Torres, Tanisha Franquez Aflague

**Affiliations:** 1 Department of Human Nutrition, Food and Animal Sciences, College of Tropical Agriculture and Human Resilience, University of Hawaiʻi at Mānoa, Honolulu, HI 96822, USA; 2 Cooperative Extension & Outreach, College of Natural and Applied Sciences, University of Guam, Mangilao, GU 96913, USA

**Keywords:** lifestyle interventions, culturally adapted, Native Hawaiian, Filipino, CHamoru, Pacific Islander

## Abstract

This research aims to describe existing evidence on the availability of culturally adapted lifestyle, nutrition, and physical activity programs among Native Hawaiian, CHamoru, and Filipino populations who are affected by obesity at rates higher than the general US population, contributing to poorer health outcomes. Addressing this disparity requires programs that are culturally adapted and grounded for these specific populations. A comprehensive description of the availability of lifestyle interventions for Native Hawaiians, Pacific Islanders, and Filipinos is missing in the literature. A systematic literature review was performed in July 2025 to gather articles that included lifestyle (nutrition and/or physical activity) interventions addressing obesity and/or related chronic diseases and that utilized one or more cultural adaptations for Native Hawaiian, CHamoru, and/or Filipino populations. Data were extracted, and methodological quality, social ecological model (SEM) level, and risk for bias was assessed. Twenty-nine articles met inclusion criteria. Interventions addressed pre-diabetes (*n* = 7), hypertension (*n* = 7), and/or obesity (*n* = 5) and included combined nutrition and physical activity (*n* = 16). Sixteen articles included interventions culturally adapted for Filipino populations only, 7 for Native Hawaiians only, 6 for both Native Hawaiians and Filipinos, and 2 included CHamorus. The most common combination of approaches were interventions that incorporated individual, interpersonal, and community SEM levels (*n* = 17). Intervention components were reflective of culturally relevant physical activities (*n* = 16) and nutrition (*n* = 11). Based on this research, there is a need for additional research to include CHamoru communities and interventions to be tested in geographic locations where these populations have migrated.

## 1. Introduction

Excessive body fat or obesity is a leading risk factor for diabetes, heart disease, stroke, and some forms of cancer. Obesity is a consequence of multiple, interwoven factors across levels of the social ecological model (SEM). The SEM can be used to describe factors across individual, interpersonal, community, organizational, and policy levels that influence diet and physical activity, which influence the risk of obesity [1]. Lifestyle programs that involve multiple SEM sectors of influence have shown promise to treat or prevent obesity and to be sustained long term [2,3,4,5]. Rates of obesity among Native Hawaiian, Filipino, and CHamoru adults are greater than White counterparts in the US and its territories [6,7,8]. This disparity may contribute to poorer health outcomes observed in Native Hawaiian, Filipino, and CHamoru populations compared to other groups [9].

Most obesity interventions have been developed and tested to serve broad populations, lacking the cultural nuances and foods unique to Native Hawaiian, CHamoru, and Filipino populations. Native Hawaiians, CHamorus, and Filipinos living in the United States (US) and territories have experienced a rapid nutrition transition over the last century, where dietary and physical activity practices have shifted because of an altered food system [10,11,12]. Furthermore, traditional Pacific Islander diets consisted predominantly of tropical fruits, vegetables, and seafood, which are not readily accessible today [13]. A recent study among CHamoru and Filipino adults in Guam revealed that foods contributing the most energy to the total diet were highly processed and lacking essential nutrients, such as white rice, sugar-sweetened beverages, fried food, and sausages [7]. Another study found that the diets of Native Hawaiian men and women were less nutritious compared to non-Hispanic Whites in the US, which is in contrast to traditional Native Hawaiian diets [14,15].

There is a wealth of evidence demonstrating that programs to improve lifestyle, diet, and physical activity can treat or prevent adult obesity [16]. There is also evidence that culturally informed programs are an effective way to develop meaningful and lasting programs among indigenous populations and minority groups that suffer disproportionately from chronic diseases related to diet and physical activity [17,18,19,20]. Native Hawaiians and CHamorus are both Pacific Islanders [21] that are indigenous to the Hawaiian Islands and the Mariana Islands—including Guam—respectively. Filipinos and CHamorus share a similar history related to the Spanish influence in both the Philippines and Guam, respectively [22,23,24]. Due to migration for agriculture and urban development, Filipinos make up a large part of the Asian population in Hawaiʻi [25], a US state, and Guam, a US territory [23,26]. Together Native Hawaiians, CHamorus, and Filipinos are grounded in similar cultural values of collectivism as well as a complex cultural history involving colonialism [27,28,29].

Connecting and reconnecting with culture for indigenous populations are emerging as key components to promote healthy lifestyle behaviors [30]. More specifically, culturally adapted and grounded approaches to program delivery involving indigenous populations have been applied to obesity, diabetes, and heart disease, yet have still not been comprehensively explored for Native Hawaiian, CHamorus, and Filipinos throughout the US in particular [31,32,33].

This systematic literature review aims to describe culturally adapted nutrition and physical activity programs designed for Native Hawaiian, CHamoru, and Filipino populations living in the US and its territories. Specifically, it describes the strategies utilized to adapt programs to be culturally appropriate for Native Hawaiian, CHamoru, and Filipino populations and identifies levels of the SEM addressed in each program.

## 2. Materials and Methods

This systematic review conforms to standardized guidelines for conducting and reporting on systematic reviews, specifically the Preferred Reporting Items for Systematic Reviews and Meta Analyses Protocols (PRISMA) guidelines [34]. A search of peer-reviewed literature was conducted in collaboration with an experienced librarian in July 2025 using the following online databases: PubMed, ProQuest, and EBSCO (CINAHL, Academic Search Complete, an Health Source: Nursing/Academic Edition). Search strategies used the same key words applied to each ethnic group, including variations of diet and physical activity programs for reducing body fat. The search terms are listed in Table 1. Screening of articles for duplicates and eligibility criteria was managed using Rayyan, a web and mobile app for systematic reviews [35]. Included articles’ bibliographies were reviewed for additional relevant and eligible articles. A total of 3 researchers were responsible for screening articles (*n* = 2), data extraction (*n* = 3), and quality appraisal (*n* = 2). Two additional researchers were consulted if there were questions about screening, data extraction, or quality appraisal.

### 2.1. Inclusion Criteria

Screening for inclusion criteria was conducted through reviews of the title, abstract, and full text, which was conducted by 2 researchers independently. Discrepancies between researchers on inclusion criteria were resolved through discussion and, in some cases, a third researcher review. The systematic review included peer-reviewed studies published in English from January 2001–July 2025. Articles were included if they employed a lifestyle (i.e., nutrition and/or physical activity) program that included 1 or more cultural adaptations and were delivered to Native Hawaiian, CHamoru, and/or Filipino populations. Both spellings of Chamorro and Chamoru were included throughout, and for this publication, the spelling CHamoru is used. Programs that focused on Pacific Islanders as an aggregated group were excluded to maintain cultural and contextual specificity. Aggregation can obscure distinct cultural and health determinants of Filipino, CHamoru, and Native Hawaiian populations, limiting the relevance and precision of findings. Quantitative and qualitative studies were included, as were experimental and quasi-experimental study designs. Programs had to include outcome measures related to diet, nutrition, exercise, or physical activity. Cultural adaptations were defined as explicitly incorporating Native Hawaiian, CHamoru, or Filipino values, norms, beliefs, and/or language in the program [36]. Cultural adaptations and sensitivity structures were assessed as surface or deep and the presence of 8 unique elements, respectively. Cultural adaptations were classified as surface level if they included minor changes, such as changing out phrases or images to better relate to the target population. Meanwhile, deep adaptations were those that were grounded in cultural values and worldviews more salient with the target population or community [37]. Cultural sensitives included 8 elements; language, persons, metaphors, content, concepts, goals, methods, and context. Language refers to the use of culturally appropriate terms; persons relates to those involved, including facilitators or peers with similar ethnic/racial backgrounds; metaphors refer to shared concepts within the population; content reflects cultural knowledge and values; concepts align with the population’s culture; goals are supportive of adaptive values from the culture of origin; methods incorporate adaptation of treatment methods; and context acknowledges cultural trauma or stress [37].

### 2.2. Data Extraction

Data from included articles were extracted and entered by 3 researchers who were trained on the review protocols. Data included the author name(s); publication year; title; database; sample size; study design; program description and length; delivery setting; geographic location of the study; outcomes measured and tools used; sex, age (mean and range), and ethnic group(s) of the study population; cultural adaptation strategies used (i.e., language, persons, metaphors, content, concepts, goals, methods, context), with the description in [37]; cultural sensitivities employed (i.e., surface or deep) [36]; SEM level(s) the program addressed (i.e., individual, interpersonal, community, organization, or policy); and main results.

### 2.3. Quality Appraisal

The Joan Briggs Institute (JBI) critical appraisal tools for randomized control trials (RCT) and quasi-experimental studies were used to assess the methodologic quality and risk of bias for all articles [38]. The JBI RCT appraisal tool includes 13 questions that assess bias related to selection and allocation of participants; administration of the program/exposure; assessment, detection, and measurement of the outcome; participant retention; as well as statistical conclusion validity measures. The JBI quasi-experimental appraisal tool includes 9 questions that assess bias related to temporal precedence of the cause and effect; selection and allocation of a control group; confounding factors; administration of the program/exposure; assessment, detection, and measurement of the outcome; participant retention; as well as statistical conclusion validity.

Two trained researchers reviewed all articles in this study and applied appropriate critical appraisal tools independently; discrepancies in scoring were resolved by an additional researcher. Each criterion was reviewed, and scores were calculated by assigning a 1 for “yes” responses and “0” for all other responses. A total score was calculated for each article (sum of scores for the criteria), and the average total score was calculated across reviewers [24]. The highest potential score for JBI RCT appraisals was 13, and the highest potential total score for the JBI quasi-experimental appraisals was 9, with a higher score indicating higher quality.

## 3. Results

**Figure 1:**
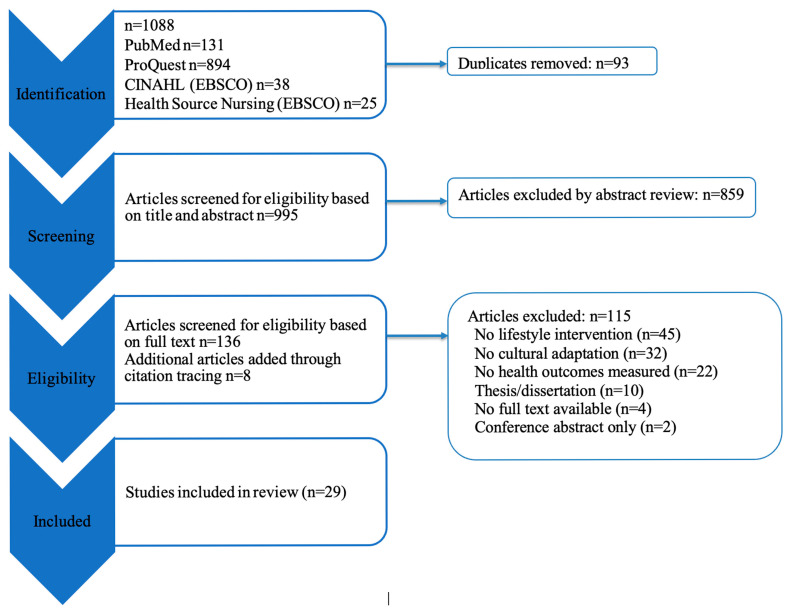
PRISMA 2020 flow diagram for systematic review.

A total of 1088 articles were identified from the database search (Figure 1). After duplicates were removed (*n* = 93), 995 articles were screened for eligibility (title and abstract). Eligible articles (*n* = 144) were screened with full-text reviews (136 identified from the original database search and 8 identified from reviews of citations). Of the 144 articles that underwent a full-text review, 29 met the inclusion criteria.

### 3.1. Characteristics of Studies Included

Table 2 contains detailed characteristics for all included articles (*n* = 29) [8,32,39,40,41,42,43,44,45,46,47,48,49,50,51,52,53,54,55,56,57,58,59,60,61,62,63,64,65]. Approximately half of the articles were published between 2016–2025 (*n* = 16) [8,32,39,40,41,44,48,49,50,51,53,58,62,63,64,65]. Most programs were designed to address hypertension (*n* = 7) [32,48,50,58,61,62,64] or pre-diabetes (*n* = 7) [39,45,47,52,53,55,56]. Fifty-five percent of programs included combined nutrition and physical activity components (*n* = 16) [8,39,40,42,43,44,45,46,47,52,54,55,56,57,59,61]. Sixteen articles included programs culturally adapted for Filipino populations only [8,32,39,40,41,42,43,45,52,53,54,61,62,63,64,65], 7 for Native Hawaiians only [44,48,49,50,51,55,58], 6 adapted for both Native Hawaiians and Filipinos [46,47,56,57,59,60], and 2 included CHamorus [37,43]. Programs primarily took place in Hawaiʻi (*n* = 15) [43,44,45,46,47,48,49,50,52,55,56,57,58,59,60], with 7 in California [8,39,40,42,51,64,65] and 7 on the East Coast of the US continent [32,41,53,54,61,62,63], with no programs delivered on Guam. 

Regarding the SEM level targeted for the programs, all programs, at a minimum, addressed the individual level, and all but 4 articles addressed additional SEM levels [54,55,60,65]. The most common combination of approaches were programs that incorporated individual, interpersonal, and community levels (*n* = 14) [8,39,40,41,43,44,48,52,53,56,58,59,61,62]. Policy level changes were only addressed by one program [32].

All programs incorporated multiple cultural adaptation strategies, with 7 articles utilizing all strategies (Table 2) [32,43,45,49,50,52,62]. The “persons” strategy was the most commonly utilized adaptation, with only 1 article not including it [60]. Meanwhile, “metaphors” were incorporated the least (*n* = 12) [32,42,43,44,45,48,49,50,52,58,59,62]. Nineteen of the programs included both surface and deep culturally sensitive adaptations [8,32,39,40,41,42,43,45,46,47,48,49,50,51,52,56,58,61,62].

Eighteen of the programs had a duration between 3 and 6 months [8,32,39,40,44,46,47,48,49,50,51,52,55,56,59,61,62,63]. The second highest frequency of duration was between 12 and 24 months (*n* = 5) [42,43,53,57,58]. Programs lasting less than 3 months were the least common (*n* = 5) [45,50,54,64,65].

### 3.2. Summary of Culturally Adapted Lifestyle Program Components

The lifestyle program components were reflective of culturally relevant physical activity and nutrition, summarized in Table 3. These included physical activity adaptations to include dance (i.e., hula, traditional Filipino dance, Zumba^®^, or cha cha) (*n* = 10) [8,39,40,41,42,48,49,50,58,63], walking (*n* = 5) [8,39,40,42,65], and gardening (*n* = 3) [32,33,34]. Regarding nutrition, the primary cultural adaptation was the inclusion of traditional and commonly consumed foods from Filipino, Native Hawaiian, and CHamoru cultures (*n* = 14) [8,32,39,40,44,47,48,52,53,56,57,59,61,64]. Many program components were developed or adapted using CBPR or community-engaged approaches (*n* = 17) [8,32,39,40,41,44,45,46,47,48,49,50,54,58,59,61,63]. Additional cultural adaptations included reliance upon trusted individuals within the community (e.g., trained peer educator, community health worker, lay leader, or community champion) to deliver the program (*n* = 21) [8,32,39,40,41,42,43,44,45,48,49,50,51,52,54,55,58,62,63,64,65], programs implemented in a familiar vernacular (e.g., native language of participants or plain or “local” language) (*n* = 26) [8,32,39,40,41,42,43,44,45,46,47,48,49,50,51,52,53,55,56,57,60,61,62,63,64,65], and/or programs delivered in settings familiar to the participants (*n* = 9) [32,41,42,44,45,52,60,61,63]. Culturally grounded programs implemented deep structure cultural sensitivity adaptations that built on shared values, such as family and collectivism (*n* = 16) [8,39,40,41,43,44,45,46,47,52,56,57,59,60,61,63].

### 3.3. Quality of Research Studies

The quality assessment scores for RCTs ranged from 2 to 10, with an average score of 7.4 and median score of 7 (*n* = 13) [8,39,40,42,45,46,48,49,50,54,57,58,59]. The quality assessment scores for quasi-experimental studies ranged from 2 to 8, with an average score of 7.3 and median score of 8 (*n* = 16) [32,41,43,44,47,51,52,53,55,56,60,61,62,63,64,65]. All 13 RCTs lacked blinding of treatment allocation [8,39,40,42,45,46,48,49,50,54,57,58,59]. A majority of quasi-experimental studies lacked control groups (*n* = 14) [32,41,43,44,47,51,53,55,60,61,62,63,64,65], yet all studies utilized reliable measures to determine impact.

## 4. Discussion

This systematic review identified a recent increase in research related to culturally adapted programs for diabetes prevention and control for Filipinos, CHamorus, and Native Hawaiians. The rising incidence and prevalence of pre-diabetes, gestational diabetes mellitus, and type 2 diabetes mellitus among Asian Pacific Islanders, which is inclusive of Filipinos, CHamorus, and Native Hawaiians, may have prompted the growth of literature [66,67,68,69]. Also noted is the disparity in literature for CHamoru adults and Native Hawaiians and Filipinos, where there continues to be gaps in programs including CHamoru adults living in Guam and across the continental US. Lastly, this systematic review found that multi-level programs and use of multiple cultural adaptation strategies was prevalent in the literature. This is in alignment with the collectivist culture of all three groups and is operationalized in the programs identified through the inclusion of family members, community organizations, and group-based activities [27].

Pre-diabetes was the most common health condition addressed by the included studies. The target populations, Native Hawaiian, Filipino and CHamoru, experience type 2 diabetes at disproportionately higher rates as compared to non-Hispanic whites; thus, primary prevention by addressing pre-diabetes has been the focus of many programs [66]. In recent years, the efforts of initiatives such as the National Diabetes Prevention Program (DPP) have led to the increased adoption of these programs as well as the development of cultural adaptations for those who experience higher risk, such as Asian and Pacific Islander populations, yet there are none for Filipinos or CHamorus [47,70,71]. Other common conditions from the systematic review included cardiovascular disease, hypertension, prehypertension, diabetes, and obesity. Lacking from the literature search results were lifestyle programs designed for persons diagnosed with cancer, cancer survivors, or chronic kidney disease, all of which disproportionately affect the target populations [72,73].

Common cultural adaptations among programs included showcasing foods commonly consumed in the target population (nutrition) and dance (physical activity). For example, many programs adapted for Filipino populations addressed frequent utilization of high sodium condiments (e.g., soy sauce and fish sauce) for hypertension control. Hula was a commonly applied adaptation to physical activity programs for Native Hawaiians. In addition, most programs included complementary cultural adaptations, such as navigating social norms and cultural expectations and the health care system resources available to participants.

The geographic locations that were represented in this study match where there are concentrated populations of the groups of interest in the US or its territories [74,75]. In addition to needing greater representation of CHamoru populations in research, more programs are needed in additional locations where these populations reside. For example, in the most recent 2020 US Census report, CHamoru was the third largest sub-group within the broader Native Hawaiian Pacific Islander population (with Native Hawaiian and Samoan being the first and second) in the US at 10.2% [25]. Filipinos were the third largest population within the Asian group in the same year [25]. In terms of where these populations reside in the US, San Diego County, California, was home to the largest CHamoru population at 27.8%. For Native Hawaiians, Honolulu County has the largest population of Native Hawaiians (77.1%) in the country, but over the past few years this has decreased by 8%, with Clark County, Nevada, and San Diego County, California, having the largest Native Hawaiian populations outside of the state of Hawaiʻi. Similarly, San Diego County, California, and Honolulu County, Hawaiʻi, had the largest populations, with Filipinos at 41.6% and 42.9% of all Asians, respectively [25]. These statistics offer a justification for expanding the development and testing of culturally adapted programs for CHamoru and Native Hawaiian populations in new areas, such as San Diego and Clark County, Nevada.

Including cultural adaptive strategies can be effective in promoting a healthy lifestyle as well as a critical strategy to preserving and perpetuating cultural practices in populations who are relocating, as is the case with Native Hawaiians [18]. Acculturation, which occurs when a population migrates and takes on the new host culture, is associated with excessive body weight, sedentary behavior, and poor diet [76,77].

The body of literature identified from this study may inform the development of programs to address other health conditions for Native Hawaiian, Filipino, and CHamorus, particularly if culturally adapted approaches are shown to be effective in improving target behavioral outcomes. For example, many programs in this study addressed interpersonal and community levels of the SEM, which is in line with other research findings that identified family (interpersonal) and community values (cross-cutting factors) to be of high importance when working with Native Hawaiian, Filipino, and CHamoru breast cancer survivors [78,79]. However, a key gap in this literature is policy level programs, which are key to long-term sustained changes in health.

Additionally, over half of the articles included relatively short program durations, between 3 and 6 months, in comparison to nationally recognized lifestyle change programs, such as the DPP, which is a 12-month program. However, a 12-week DPP adapted for Native Hawaiians, Partners in Lifestyle Programs Ohana Lifestyle Program, yielded significant weight loss results among pilot study participants, despite being delivered in a reduced length of time, which was found to be more desirable for the Native Hawaiian community members [57]. Focus groups conducted with Native Hawaiian, Filipino, and CHamoru breast cancer survivors similarly found that a 10-week lifestyle program would be most desirable [55].

To the authors knowledge, this is the first systematic literature review conducted that aims to describe the availability of high quality, culturally adapted, lifestyle programs for Native Hawaiians, CHamorus, and Filipinos. The reliance on PRISMA guidelines is a strength of this study, as is the utilization of multiple reviewers in extracting data and assessing quality. A key limitation of this work is the subjective nature of classifying the cultural adaptations. The authors overcame this through training reviewers, using published definitions for consistency, and a classification process which relied upon independent reviewers; nonethless, some of the articles lacked detailed descriptions of the cultural adaptations, which made the process difficult for reviewers. In addition, this review did not aim to describe the effectiveness of the programs included. A future meta-analysis might aid in strengthening the evidence on the impact of these culturally adapted programs.

## 5. Conclusions

Findings from this review can be used to design effective and relevant health and lifestyle programs for these populations in communities where Native Hawaiians, Filipinos, and CHamorus reside. More studies including CHamoru communities and that are conducted in geographic locations where populations in this study are living are warranted. Programs that include cultural values and practices of communities are needed to elicit sustainable lifestyle changes to address health disparities.

## Figures and Tables

**Table 1:** Search terms by database for systematic review on cultural adaptations for lifestyle interventions for Native Hawaiian, CHamoru, and Filipino adults.

Database	Search Terms
PubMed	Search 1: (intervention AND (“diet” OR “nutrition” OR “physical activity” OR “exercise” OR “lifestyle”)) AND cultur* AND Filipin* Search 2: (intervention AND (“diet” OR “nutrition” OR “physical activity” OR “exercise” OR “lifestyle”)) AND cultur* AND (CHamor* OR “Guam”) Search 3: (intervention AND (“diet” OR “nutrition” OR “physical activity” OR “exercise” OR “lifestyle”)) AND cultur* AND “Native Hawaiian”
ProQuest	Search 1: noft(“intervention” AND (“diet” OR “nutrition” OR “physical activity” OR “exercise” OR “lifestyle”)) AND cultur* AND Filipin* Search 2: noft(“intervention” AND (“diet” OR “nutrition” OR “physical activity” OR “exercise” OR “lifestyle”)) AND cultur* AND (CHamor* OR “Guam”) Search 3: noft(“intervention” AND (“diet” OR “nutrition” OR “physical activity” OR “exercise” OR “lifestyle”)) AND cultur* AND “Native Hawaiian” To narrow search: used noft = no full text
EBSCO (CINAHL, Academic Search Complete, and Health Source: Nursing/Academic Edition)	Search 1: (intervention AND (“diet” OR “nutrition” OR “physical activity” OR “exercise” OR “lifestyle”)) AND cultur* AND Filipin* Search 2: (intervention AND (“diet” OR “nutrition” OR “physical activity” OR “exercise” OR “lifestyle”)) AND cultur* AND (CHamor* OR “Guam”) Search 3: (intervention AND (“diet” OR “nutrition” OR “physical activity” OR “exercise” OR “lifestyle”)) AND cultur* AND “Native Hawaiian”

**Table 2:** Characteristics of studies included in the systematic review on cultural adaptations for lifestyle programs for Native Hawaiian, CHamoru, and Filipino adults.

Included Studies Author Name (Publication Year)	Focus	Program Type			Ethnic Group Included				Location			Social Ecological Model (SEM)					Cultural Sensitivity Elements								Cultural Adaptation	
	Health Condition ^1^	Nutrition	Physical Activity	Other lifestyle	Native Hawaiian	Filipino	CHamoru	Pacific Islander, Undefined	Hawaiʻi	California	East Coast	Individual	Interpersonal	Community	Organizational	Policy	Language	Persons	Metaphor	Content	Concepts	Goals	Methods	Context	Deep	Surface
Bender et al. (2017) [40]	DM, CVD	×	×			×				×		×	×	×			×	×		×	×	×	×	×	×	×
Bender et al. (2018) [39]	OB, Pre-DM	×	×			×				×		×	×	×			×	×		×	×	×	×	×	×	×
Bhimla et al. (2018) [63]	CD		×			×					×	×	×		×		×	×		×	×	×	×	×		×
Bhimla et al. (2021) [41]	CD		×			×					×	×	×	×			×	×		×	×	×	×	×	×	×
Dirige et al. (2013) [42]	HP	×	×			×				×		×	×		×			×	×	×	×	×	×	×	×	×
Fernandes et al. (2012) [43]	CVD	×	×			×			×			×	×	×			×	×	×	×	×	×	×	×	×	×
Ho-Lastimosa et al. (2019) [44]	HP	×	×	×	×				×			×	×	×			×	×	×	×	×	×				×
Inouye et al. (2014) [45]	Pre-DM	×	×	×		×			×			×	×	×	×		×	×	×	×	×	×	×	×	×	×
Kaholokula et al. (2012) [46]	OB	×	×	×	×	×		×	×			×	×	×	×		×	×		×	×	×	×	×	×	×
Kaholokula et al. (2014) [47]	Pre-DM	×	×		×	×		×	×			×	×				×	×		×	×	×	×	×	×	×
Kaholokula, Look, Mabellos et al. (2017) [48]	HTN		×		×			×	×			×	×	×			×	×	×	×	×		×	×	×	×
Kaholokula, Look, Wills et al. (2017) [49]	CVD		×		×				×			×	×				×	×	×	×	×	×	×	×	×	×
Kaholokula et al. (2021) [50]	HT, CVD		×		×				×			×	×				×	×	×	×	×	×	×	×	×	×
Kwon et al. (2017) [32]	HP, HTN	×		×		×					×	×	×	×	×	×	×	×	×	×	×	×	×	×	×	×
LaBreche et al. (2016) [51]	HP, OB		×		×		×	×		×		×		×	×		×	×		×	×	×	×	×	×	×
Leake et al. (2012) [52]	Pre-DM	×	×	×		×			×			×	×	×			×	×	×	×	×	×	×	×	×	×
Ma et al. (2019) [53]	HP, Pre-DM, Pre-CVD, Pre-HTN	×				×					×	×	×	×			×	×		×			×	×		×
Ma et al. (2021) [54]	Pre-HTN	×	×	×		×					×	×						×		×			×	×		×
Maglalang et al. (2017) [8]	DM	×	×	×		×				×		×	×	×			×	×		×	×		×	×	×	×
Mau et al. (2001) [55]	Pre-DM	×	×	×	×				×			×						×					×			×
Mau et al. (2010) [56]	OB, Pre-DM	×	×	×	×	×		×	×			×	×	×			×	×		×	×	×	×		×	×
Nguyen et al. (2024) [65]	HP		×			×				×		×					×	×		×			×			×
Novotny et al. (2012) [57]	OB	×	×		×	×	×	×	×			×	×		×					×						×
Railey et al. (2022) [58]	HTN		×		×				×			×	×	×				×	×	×	×	×	×	×	×	×
Sijangga et al. (2023) [64]	HTN	×				×				×		×	×				×	×		×	×	×	×	×	×	
Sinclair et al. (2013) [59]	DM	×	×	×	×	×		×	×			×	×	×			×	×	×	×						×
Tomioka et al. (2014) [60]	DM		×	×	×	×		×	×			×					×	×			×	×	×			×
Ursua et al. (2014) [61]	HTN	×	×			×					×	×	×	×			×	×		×	×			×	×	×
Yi et al. (2019) [62]	HTN	×		×		×					×	×	×	×			×	×	×	×	×	×	×	×	×	×
Total	-	20	25	12	13	22	2	8	15	7	7	29	24	18	7	1	24	28	12	27	23	20	25	22	20	28

^1^ CD = Chronic Disease, CVD = Cardiovascular Disease, DM = Diabetes Mellitus, HP = Health Promotion, HTN = Hypertension, Pre-CVD = Risk of Cardiovascular Disease, Pre-DM = Pre-diabetic, Pre-HTN = Risk of Hypertension, and OB = Obesity.

**Table 3:** Descriptions of cultural adaptations and results of studies included in the systematic review on cultural adaptations for lifestyle programs for Native Hawaiian, CHamoru, and Filipino adults.

Included Studies’ Author Name (Publication Year)	Nutrition Cultural Adaptation	Physical Activity Cultural Adaptation	Language Adaptation	Sociocultural and Community-Engaged Components
Bender et al. (2017) [40]	Photos of common Filipino foods were used in Filipino food pamphlets. Healthy Filipino food alternatives and recipes shared.	Educational materials tailored for Filipino cultural values and beliefs related to physical activity. Culturally relevant activities, such as Zumba^®^, cha cha, basketball, and walking.	Healthy lifestyle education pamphlets translated in Tagalog for Filipino Americans.	Family members were welcome to attend in-office visits. Community stakeholders informed the study design and trained community health workers from Filipino communities to deliver the programs. Informed by past studies among Filipinos.
Bender et al. (2018) [39]	Photos of common Filipino foods were used in Filipino food pamphlets, with healthier options as substitutes for commonly eaten unhealthy Filipino foods and drinks.	Photos of Filipino family members exercising and walking together outside and Filipino activities—walking, dancing, Zumba^®^, basketball, and bowling. Indoor options to limit sun exposure.	English and Tagalog utilized to deliver lessons.	Family members were welcome to attend in-office visits. Filipino community health workers trained to deliver program.
Bhimla et al. (2018) [63]	None.	Zumba^®^ held at a Filipino community center and local church near participants’ homes.	Bilingual Filipino instructors.	Informed by community leaders and female community members. Filipino community health workers were trained to deliver program. Group fitness classes were designed to enhance collectivism.
Bhimla et al. (2021) [41]	None.	Culturally relevant physical activity classes: Zumba^®^, line dance, Hip Hop, and strength training in a group setting.	Facilitators spoke two Filipino dialects. English and Tagalog were utilized to deliver lessons.	Informed by past research on preferred activities for this population. Recruitment involved Catholic churches frequented by Filipino families. Family- and community-oriented. Filipino community health workers were trained to deliver program.
Dirige et al. (2013) [42]	Hands-on activities.	Group activities (e.g., aerobic classes, kickboxing, dancing, gardening, and basketball tournaments).	Surveys were available in Tagalog and administered by bilingual staff.	Program was conducted through Filipino social clubs (i.e., Filipino–American social clubs in San Diego, CA, USA).
Fernandes et al. (2012) [43]	Culturally tailored curriculum lessons.	Community gardening and chair aerobics.	Nutrition booklets were provided in Tagalog. Community health workers spoke Tagalog.	Recruitment from a Filipino neighborhood. Community health workers were experienced in working with this community, led physical activities, and planned monthly celebrations with families.
Ho-Lastimosa et al. (2019) [44]	Use of Hawaiian cultural practices and food sovereignty; la’au lapa’au (Native traditional healing through plants and spirituality); ’ai pono (nourishing foods); and cultural value of ’aina (land) and malama ’aina (taking care of the land).	Gardening (i.e., planting, growing, and harvesting plants).	Incorporation of olelo Hawaiʻi (Hawaiian language) into program materials.	Built on previous community grassroots efforts related to backyard aquaponics. Workshops that used a hands-on family-based collective learning approach, which aligns with Native Hawaiian educational pedagogy, took place in a Native Hawaiian community place. Native Hawaiian cultural practices emphasized.
Inouye et al. (2014) [45]	Not described in detail.	Not described in detail.	Facilitators fluent in English and two Filipino dialects.	Recruitment from Catholic churches frequented by Filipino families. Filipino health care workers served as small group leaders. Family invited to small group sessions. Philippine Nurses Association members served as the advisory committee during the curriculum design, recruitment, and evaluation.
Kaholokula et al. (2012) [46]	Family meal planning exercise.	Family physical activity planning. Scheduling of free time for family activities.	Sessions delivered in a native language by a bilingual community healthcare and peer educator.	Strategies for each session were identified by community assessments and input from community investigators. Sessions incorporated family and cultural community activities.
Kaholokula et al. (2014) [47]	Locally relavant food sources.	None.	Program delivered in language native to the Chuukese group.	Informed by community assessments among Native Hawaiians and Pacific Islanders. Lessons were delivered in group settings to emphasize the cultural value of ’ohana (i.e., the preference for working together and group decision making). Lessons were made practical for the socioeconomic realities of many Native Hawaiians and Pacific Islanders.
Kaholokula, Look, Mabellos et al. (2017) [48]	Cooking demonstrations of healthy recipes of relavant ethnic foods.	Hula, Native Hawaiian cultural dance utilized and led by kumu hula (hula expert).	Olelo Hawaiʻi (Hawaiian language) was utilized during lessons.	Community-based participatory research (CBPR) approach informed study. Involved Native Hawaiian and Pacific Islander community investigators.
Kaholokula, Look, Wills et al. (2017) [49]	None.	Hula, Native Hawaiian cultural dance.	Olelo Hawaiʻi (Hawaiian language) was utilized during lessons.	Community-based participatory research (CBPR) approach informed study. Classes led by kumu hula (hula expert) and relevant peer educators.
Kaholokula et al. (2021) [50].	None.	Hula, Native Hawaiian cultural dance utilized and led by kumu hula (hula expert).	Olelo Hawaiʻi (Hawaiian language) was utilized during lessons.	Community-based participatory research (CBPR) approach informed study. Involved Native Hawaiian and Pacific Islander community investigators.
Kwon et al. (2017) [32]	Nutrition outreach materials tailored to address Asian condiments and sources of sodium. Incorporated fruits and vegetables such as bitter melon, cabbage, guava, starfruit, or healthy traditional foods/modifications in program materials.	None.	Surveys were translated (i.e., into Tagalog) and administered by bilingual staff.	Program guided by Filipino communities and local health departments. Pastors were enrolled in study and disseminated project info through faith-based wide announcements in Filipino churches.
LaBreche et al. (2016) [51]	None.	Physical activity video incorporating movements, cultural elements, people, and music from Pacific Islands and collaboration with a Pacific Islander (PI) filmmaker.	National Cancer Institute cancer prevention posters translated into Pacific Islander languages, such as Chamoru, Fijian, Marshallese, Samoan, and Tongan.	Program delivered by community leaders and focused on Pacific Islander social, cultural, and faith-based organizations.
Leake et al. (2012) [52]	Filipino cultural foods.	Filipino cultural activities.	Bilingual facilitator. Curriculum was presented in English interspersed with Tagalog. Filipino proverbs were incorporated into lessons.	Facilitated by a community member (Filipino leader). Delivered in an area frequented by Filipino–American families, also located near two churches. Scheduling accounted for family, social, and work obligations.
Ma et al. (2019) [53]	Nutrition messages included cultural foods and Asian food markets.	None.	Bilingual translators. Education materials were in English and Asian ethnic languages (Filipino).	Communication via Asian newspapers and media outlets. Technical assistance provided to community-based organizational support staff.
Ma et al. (2021) [54]	None.	Culturally appropriate physical activity sessions	None.	Sessions were led by Filipino community health educators. Community-based participatory research (CBPR) framework was used to guide the development of the study program.
Maglalang et al. (2017) [8]	Photos of common Filipino foods were used in Filipino food pamphlets. Healthy Filipino food alternatives and recipes shared.	Culturally relevant activities, such as Zumba^®^, cha cha, basketball, and walking. Relapse prevention for Filipino American sedentary cultural practices (e.g., extended Mahjong playing time).	Healthy lifestyle education pamphlets translated in Tagalog for Filipino Americans.	Family members were welcome at in-office visits. Community stakeholders informed study design and trained community health workers from Filipino communities to deliver the programs. Informed by past studies among Filipinos.
Mau et al. (2001) [55]	None.	None.	Incorporation of olelo Hawaiʻi (Native Hawaiian language) into program (example: ʻohana for family).	Culturally responsive lifestyle program was developed and implemented by trained community peer educators.
Mau et al. (2010) [56]	Local food examples and common nutrition and diet-related behaviors.	Group-based classes, in line with a collectivist cultural value.	The focus groups were conducted in a language native to Chuukese, Filipino, and Samoan groups. Lessons were in “plain language”, with cultural/linguistic relevance to Native Hawaiians and Pacific Islanders.	Program was designed using community-based participatory research (CBPR). Program was delivered by peer educators. Social support issues related to cultural practices were addressed.
Nguyen et al. (2024) [65]	None.	App had visuals adapted to the preferred cutural context and delivered messages in a tone and format familiar to each group.	Program was adapted for Chinese, Tagalog, and Vietnamese speakers. All program materials were translated, followed by critical reviews of cultural and language equivalence at a 4th-grade reading level by culturally and linguistically competent staff.	Worked with trusted ethnic community organizations. Staff were bicultural.
Novotny et al. (2012) [57]	Ethnic foods commonly consumed by the general population in Hawaiʻi were emphasized.	Physical activities appropriate for the local environment were incorporated. Group-based classes were designed.	Surveys were available in English, Chinese, and Korean.	Program was designed around the broad patterns of culture found in Hawaiʻi due to multiethnic participants.
Railey et al. (2022) [58]	None.	Hula—traditional Hawaiian dance.	None.	Programs were delivered by community members (Kumu hula, or hula expert, and peer educators). Community-based organizations were involved in the study design and interpretation of findings.
Sijangga et al. (2023) [64]	Traditional Filipino recipes were adapted to be low sodium, fat, and cholersterol while maintaining traditional flavors; use of Filipino ingredients, cookbook created with Filipino chefs, story telling framework in cookbook.	None.	Tagalog was used in the title of the cookbook.	Cultural practice of shared meals with family and friends. Carefully selected individuals (Filipino American culinary experts) were chosen to contribute to cookbook recipes.
Sinclair et al. (2013) [59]	Images of Hawaiʻi and local foods.	Images of physical activity relevant to Hawaiian environment were included.	“Local” language and examples were used to convey some educational content.	Community leaders and health advocates from four distinct community organizations serving Native Hawaiians and Pacific Islanders. Program theory included cultural symbols and themes, cultural patterns and concepts, values, norms, and relationships. Group-based educational format was used to facilitate social support. Storytelling utilized locally relevant examples of personal experiences with diabetes.
Tomioka et al. (2014) [60].	None.	None.	Bilingual trained staff who took time during and after each session to reinforce key messages in participants’ native languages.	Recruitment was held at community events, such as health fairs and word-of-mouth recruitments from previous participants. Pre-workshop orientation by program leaders and a community physician. Involved family members at a graduation party, 6-month “reunion”, and follow up.
Ursua et al. (2014) [61]	Food examples represented foods availabile within the community,	Exercise examples represented activities availabile within the community.	Program was delivered by bilingual Filipino community health workers.	Curriculum was culturally designed for Filipino American community through community-engaged process. Programs held at the local library, community centers, apartment buildings, and the lead community partner’s office. Incoporated Filipino history and culture and health and social services and health insurance options.
Yi et al. (2019) [62]	Culturally tailored lifestyle counseling on weight management; examples of healthy plates using common cultural foods.	None.	Surveys were translated (i.e., into Tagalog) and administered by bilingual staff.	Trained faith-based leaders implemented the culturally adapted program for four Asian American communities.

## Data Availability

Data is available upon request.
